# Lateral lymph node dissection reduces local recurrence of locally advanced lower rectal cancer in the absence of preoperative neoadjuvant chemoradiotherapy: a systematic review and meta-analysis

**DOI:** 10.1186/s12957-020-02078-1

**Published:** 2020-11-23

**Authors:** Xiang Gao, Cun Wang, Yongyang Yu, Dujanand Singh, Lie Yang, Zongguang Zhou

**Affiliations:** 1 Institute of Digestive Surgery, Sichuan University, Chengdu, Sichuan China; 2 Department of Gastrointestinal Surgery, West China Hospital, West China School of Medicine, Sichuan University, Chengdu, Sichuan China

**Keywords:** Rectal cancer, Lateral lymph node dissection, Total mesorectal excision, Neoadjuvant chemoradiotherapy

## Abstract

**Background:**

The impact of lateral lymph node dissection (LLND) in locally advanced lower rectal cancer remains controversial. This study is to compare total mesorectal excision (TME) with or without LLND in lower rectal cancer cases of stage II/III.

**Methods:**

The electronic databases were systematically searched that compared TME with or without LLND among patients with lower rectal cancer in clinical stage II/III. Subgroup analysis was performed considering neoadjuvant chemoradiotherapy (nCRT). The hazard ratios (HR), relative risk (RR), and weighted mean difference (WMD) were pooled.

**Results:**

Twelve studies of 4458 patients of this meta-analysis demonstrate, LLND alone significantly reduced the local recurrence rate of patients who did not receive nCRT (RR 0.71, *P* = 0.004), while the difference was not significant when combined with nCRT (RR 0.70, *P* = 0.36). The analysis shows TME with LLND was associated with significantly longer operation time (WMD 90.73 min, *P* < 0.001), more intraoperative blood loss (WMD 303.20 mL, *P* < 0.001), and postoperative complications (RR = 1.35, *P* =0.02). Whereas urinary dysfunction (RR 1.44, *P* = 0.38), sexual dysfunction (RR 1.41, *P* = 0.17), and postoperative mortality (RR = 1.52, *P* = 0.70), were similar between these two groups. Statistically, no significant differences were observed in OS (HR 0.93, *P* = 0.62), DFS (HR 0.99, *P* = 0.96), total recurrence (RR 0.98, *P* = 0.83), lateral recurrence (RR 0.49, *P* = 0.14), or distal recurrence (RR 0.95, *P* = 0.78) between these two groups regardless of whether nCRT was performed or not.

**Conclusions:**

The study shows LLND alone decreases the local recurrence without using nCRT irrespective of the survival advantage in locally advanced lower rectal cancer. The benefit of controlling local recurrence by LLND alone makes us reconsider the usage of nCRT with LLND.

**Trial registration:**

The protocol for this meta-analysis was registered prospectively with PROSPERO (CRD42020135575) on 16 May 2019.

**Supplementary Information:**

The online version contains supplementary material available at 10.1186/s12957-020-02078-1.

## Background

The total mesorectal excision (TME) technique has significantly improved the pathological and oncological outcomes and has become the standard surgical procedure for rectal cancer. Approximately 14–30% of patients with lower rectal cancer in clinical stage II/III develop pelvic lateral lymph node (LLNs) metastases, which is beyond the surgical field of TME and is associated with an increased incidence of local recurrence and decreased survival [1, 2]. In Japan, lateral lymph node dissection (LLND) has been recommended as the standard treatment for patients with lower rectal cancer in clinical stage II/III since the 1970s [3, 4]. On the other hand, Western surgeons believe, LLNs metastases were a sign of distant metastasis and cannot be eliminated by surgery. Therefore, preoperative nCRT instead of LLND has become the standard regimen for the treatment of locally advanced lower rectal cancer in Western countries [5, 6]. However, studies have shown that preoperative nCRT could not completely eradicate the metastatic LLNs, suggesting nCRT followed by TME and LLND may be more effective in the management of locally advanced lower rectal cancer [7, 8].

The efficiency and safety of LLND in locally advanced lower rectal cancer remain controversial in studies. Some studies have revealed LLND could considerably reduce the local recurrence of patients with rectal cancer and improve survival [9, 10]. Where other studies showed LLND has no benefits in improving survival or reducing recurrence rates. Additionally, it increases urinary and sexual dysfunction [11, 12]. Two previous meta-analyses performed approximately 10 years ago indicated LLND had no advantage in controlling recurrence or improving survival and appeared to be associated with increased urinary and sexual dysfunction [13, 14]. However, neither of these two meta-analyses explicitly restricted tumor anatomical location and clinical stage, leading to upper rectal cancers and early-stage rectal cancers were included in their studies. Actually, the application of LLND in upper-third or early-stage rectal carcinoma has practically been abandoned since 2000, and LLND was primarily performed for locally advanced lower rectal cancer at present [4]. In addition, nCRT is currently the primary treatment regimen for locally advanced rectal cancer, and neither of these two meta-analyses separately assessed the effects of LLND on patients who have received preoperative nCRT.

Studies, including large RCTs and well-designed cohorts, performed earlier to clarify the significance of LLND in stage II/III lower rectal cancer, although could not provide clear results [9, 15, 16]. Thus, this meta-analysis is an attempt to integrate the outcomes of previous studies. It assesses the efficacy and safety of LLND in locally advanced lower rectal cancer with or without nCRT which is remained controversial. The protocol for this meta-analysis was registered prospectively with PROSPERO (CRD42020135575).

## Methods

### Literature search

A systematic search of all peer-reviewed literature was performed in electronic databases, including MEDLINE (via PubMed), Embase, Ovid, and the Cochrane Library up to 22 December 2019. The literature of the Google Scholar database was also reviewed. The following MeSH search headings and their synonyms were used: “total mesorectal excision,” “lateral lymph node dissection,” “extended lymphadenectomy,” “lateral pelvic wall lymph-node dissection,” “rectal neoplasms,” “rectal cancer,” “comparative study,” and “treatment outcome.” The related-articles were used to broaden the search. The reference lists of relevant studies and systematic reviews were screened manually. A full-text review was performed after a screening of the title and abstract. The data were extracted based on criteria framed.

### Selection criteria

All comparative studies evaluating the efficiency or safety of TME combined with LLND versus TME alone in the treatment of stage II/III lower rectal cancer were included. Studies with the following inclusion criteria were eligible: (1) patients with locally resectable clinical stage II/III rectal cancer without evidence of distal metastasis and tumor location within 8 cm from the anal verge, or the major part of tumor located at or below the peritoneal reflection; (2) Patients between the two groups with similar clinical characteristics and therapeutic protocols. The following exclusion criteria were used: (1) tumor lesions located in the upper third of the rectum, or the major part of tumor located above the peritoneal reflection; (2) patients with significantly different clinical characteristics between the two groups; (3) patients with distant metastasis at the time of treatment or other malignant diseases or fixed tumors; and (4) animal studies, letters, comments, and editorials. In cases of the considerable overlap in subjects between studies published on a single clinical trial, the most recent or most informative study was included, and the results were used complementary.

### Data extraction

Two reviewers independently performed data extraction and study quality assessment. Consistent extraction data, between reviewers were used directly for the final analysis. Disagreements between reviewers were discussed and resolved via consensus. The primary endpoints were 5-year overall survival (OS) and disease-free survival (DFS). Secondary endpoints including total recurrence, local recurrence, lateral recurrence, distant recurrence, operation time, intraoperative blood loss, postoperative complications and mortality, urinary dysfunction, and sexual dysfunction.

### Study quality assessment

The Newcastle-Ottawa scale criteria recommended by the Cochrane Library for including trials were used to evaluate the quality of the cohort studies. The quality of RCTs was measured by using the Cochrane Collaboration's risk for a bias assessment tool. Two reviewers assessed the quality of the studies. Where discrepancies arose, papers were re-examined, and the consensus was reached via discussion.

### Statistical analysis

The meta-analysis is based on Cochrane Collaboration and the Quality of Reporting of Meta-analyses (QUORUM) guidelines [17, 18]. Hazard ratios (HRs) and the respective 95% confidence intervals (CIs) were assessed as effective measures for time-to-event data (5-year OS and DFS). In the absence of HR information, we used the estimation of data from other given information (e.g., Kaplan-Meier plots) [19, 20]. Risk ratio (RR) was used as the summary statistic for statistical analyses of dichotomous variables, and weighted mean difference (WMD) was used to analyze continuous variables. P-values for the overall effects were calculated based on a two-sided *Z*-test for independent samples for effective measures on a log scale. A *P* value < 0.05 was considered statistically significant. Meta-analytic results were graphically displayed in Forest plots. Heterogeneity was tested using chi-squared analyses and defined as present in cases of a *P* value < 0.10. *I*^2^ > 40% was considered statistically significant heterogeneity, and the random-effects model was used to calculate overall effect estimates after examining the causes of heterogeneity. Otherwise, the fixed-effects model was used. Subgroup analysis was performed based on whether preoperative nCRT was undertaken. Review Manager version 5.3 was used for the meta-analysis (Copenhagen, the Nordic Cochrane Centre) [21].

## Results

### Study selection

**Fig. 1:**
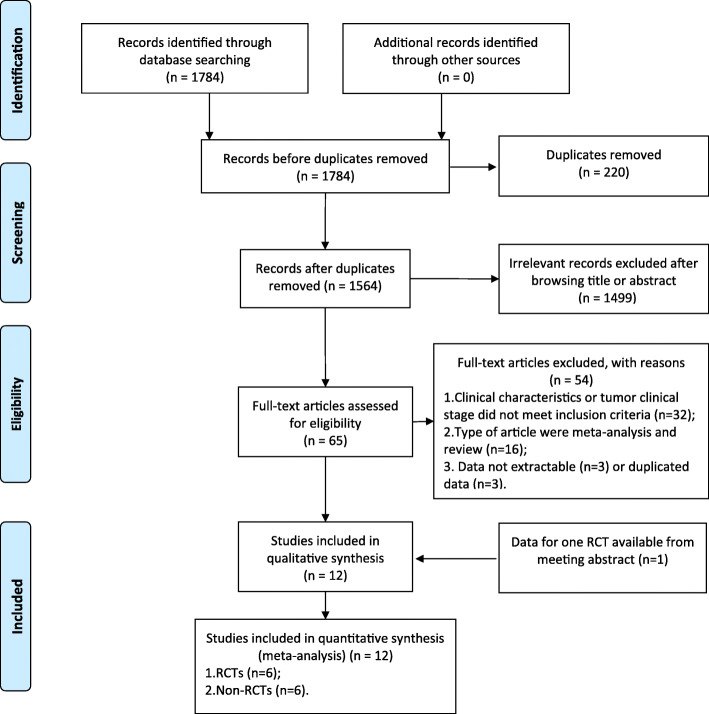
PRISMA flow chart

A total of 1564 citations were identified using the predefined search strategy (Fig. 1). After screening the titles and abstracts, 1499 of the studies were excluded due to lack of relevance. Sixty-five articles were further evaluated for eligibility. Among these publications, 54 studies were excluded due to the following reasons: 32 studies did not meet selection criteria; 2 studies were meta-analyses; 14 studies were reviews; 3 studies data were not extractable; 3 studies with overlapping data. Four studies based on one same randomized trial were included because they reported different outcomes. Full manuscripts were available for 11 studies, and the results of one RCT were available as a conference proceeding presented on the 2017 ECCO European Cancer Congress [15]. Finally, twelve studies published from 2001 to 2019 and involving a total of 4458 patients (1952 in the TME + LLND group and 2506 in the TME alone group) fulfilled the selection criteria were included in the current meta-analysis. The flow diagram is shown in Fig. 1.

### Characteristics of the included studies

Six studies were RCTs, and the remaining six studies were non-RCTs. According to the Cochrane bias assessment, all of the RCTs mentioned “randomization,” but only four studies (based on the same research) reported as an adequate randomized sequence and mentioned that the allocation procedure was not masked to investigators or patients. Another two RCTs failed to report the randomization procedure or mentioned whether blinding was adopted (please refer. Additional file 1 and Additional file 2). The six non-RCTs were all cohort studies, including five retrospective studies and one prospective study with prospectively collected data. The quality of the non-RCTs was evaluated using the Newcastle-Ottawa criteria. As shown in Table 1, the total number of stars of the six non-RCTs was not less than seven for each study. The basic information about the eligible studies is listed in Table 2. Study outcomes are shown in Table 3.

**Table 1:** Scores of 6 cohort studies using Newcastle-Ottawa Criteria

Study	Selection				Comparability	Outcomes			Total
	Representativeness of the exposed cohort	Selection of the non-exposed cohort	Ascertainment of exposure	Demonstration that outcome of interest was not present at the start of the study	Comparability of cohorts on the basis of the design or analysis	Assessment of outcome	Was follow-up long enough for outcomes to occur	Adequacy of the follow-up of cohorts	
Fujita, S. 2003 [2]	1	1	1	1	0	1	1	1	7
Kusters, M. 2009 [6]	1	1	1	1	2	0	1	1	8
Watanabe, T. 2002 [22]	1	1	1	1	1	1	1	1	8
Oki, Eiji 2019 [12]	1	1	1	1	1	1	1	1	8
Ozawa, H. 2016 [10]	1	1	1	1	2	0	1	1	8
Ogura 2019 [16]	1	1	1	1	0	1	1	1	7

**Table 2:** Characteristics of the 12 included studies

Study	Study year(region)	Research type	Group name	Sample size	Mean age(years)	Sex ratio(M:F)	Median follow-up time	Tumor location	Clinical stage	Preoperative therapy regimen (***n***)	Postoperative therapy regimen (***n***)	Indication of LLND	Type of LLND	Type of TME	Matching criteria
Nagawa, H	2001(Japan)	RCTs	TME+LLND	23	59.1(± 10.1)^b^	17:6	N/A	Middle, low	Stage B, C ^Ω^	50Gy (2 Gy/day*5 days/week*5 weeks) (23)	5-FU-based chemotherapy (23)	Random controlledallocation	Bilateral LLND	LAR, APR	A, B, C, D, E, F, H, I
			TME alone	22	60.1(± 8.8)^b^	16:6				50 Gy (2 Gy/day*5 days/week*5 weeks) (22)	5-FU-based chemotherapy (22)		No		
Fujita, S	2003(Japan)	Retrospective	TME+LLND	204	57(± 10)^b^	133:71	59 months	Middle, low	TNM II/III	No	No	No lateral lymph nodes metastases	Bilateral LLND	LAR, APR	A, B, C, D, E, F, G, H, I
			TME alone	42	64(± 12)^b^	24:18							No		
Kusters, M	2009(Japan and Netherlands)	Retrospective	TME + LLND	324	58(± 11)^b^	215:109	7.9 years	Middle, low	TNM II/III	No	Postoperative chemoradiotherapy (27)	Non-restriction but the same inclusion criteria in the two arms	Unilateral and bilateral LLND	LAR, APR	B, C, D, E, F, G, I
			TME alone	376	64(± 11)^b^	234:142	7.0 years				Postoperative chemoradiotherapy (61)		No		
Watanabe, T	2002(Japan)	Retrospective	TME + LLND	75	N/A	N/A	N/A	Middle, low	Stage B, C ^Ω^	50 Gy(2 Gy/day*5 days/week*5 weeks) (75)	No	No lateral lymph nodes metastases	Bilateral LLND	LAR, APR, or Hartmann	A, B, C, D, E, G, H, I
			TME alone	40	N/A	N/A				50 Gy(2 Gy/day*5 days/week*5 weeks) (40)			NO		
Fujita, S	2012(Japan)	RCTs	TME + LLND	351	61(54–67)^a^	236:115	N/A	Middle, low	TNM II/III	No	5-FU-based chemotherapy (163)	Random controlledallocation	Bilateral LLND	LAR, APR, or Hartmann	A, B, C, D, E, F, G, H, I
			TME alone	350	62(55–68)^a^	236:114					5-FU-based chemotherapy (153)		NO		
Dev, K	2017(India)	RCT	TME + LLND	163	N/A	N/A	N/A	Middle, low	TNM II/III	25 Gy(5 Gy*5) (163)	NO	Random controlledallocation	Bilateral LLND	TME without explaining the details	A, B, C, D, E, F, G, H, I
			TME alone	77	N/A	N/A				25 Gy(5 Gy*5) (77)			NO		
Saito, S	2016(Japan)	RCTs	TME + LLND	351	61(55–66)^a^	N/A	N/A	Middle, low	TNM II/III	No	5-FU-based chemotherapy (163)	Random controlledallocation	Bilateral LLND	LAR, APR, or Hartmann	A, B, C, D, E, F, G, H, I
			TME alone	350	62(56–69)^a^	N/A					5-FU-based chemotherapy (153)		NO		
Fujita, S	2017(Japan)	RCTs	TME + LLND	351	61(26–75)^@^	236:115	72.2 months	Middle, low	TNM II/III	No	5-FU-based chemotherapy (163)	Random controlledallocation	Bilateral LLND	LAR, APR, or Hartmann	A, B, C, D, E, F, G, H, I
			TME alone	350	62(26–75)^@^	236:114					5-FU-based chemotherapy (153)		NO		
Ito, Masaaki	2018(Japan)	RCTs	TME + LLND	351	61(26–75)^@^	236:115	N/A	Middle, low	TNM II/III	No	5-FU-based chemotherapy (163)	Random controlledallocation	Bilateral LLND	LAR, APR, or Hartmann	A, B, C, D, E, F, G, H, I
			TME alone	350	62(26–75)^@^	236:114					5-FU-based chemotherapy (153)		NO		
Oki, Eiji	2019(Japan)	Prospective	TME + LLND	215	60.7(± 9.4)^b^	159:56	5 years	Middle, low	TNM II/III	No	5-FU-based chemotherapy (215)	Random controlledallocation	Bilateral LLND	LAR, APR, Hartmann, or Others	B, C, D, E, F, G, H, I
			TME alone	230	63.5(± 8.9)^b^	151:79					5-FU-based chemotherapy (230)		NO		
Ozawa, H	2016(Japan)	Retrospective	TME+LLND	499	N/A	356:143	N/A	Middle, low	TNM II/III	No	Postoperative chemotherapy (193)	Non-restriction but the same inclusion criteria in the two arms	Bilateral LLND	LAR, APR, or Others	A, B, C, D, E, F, I
			TME alone	499	N/A	334:165					Postoperative chemotherapy (207)		NO		
Ogura	2019(Japan)	Retrospective	TME+LLND	98	N/A	N/A	56.5 (55.0–58.1) ^@^	Middle, low	TNM II/III	45-50.4 Gy/25 Gy + oxaliplatin-based/5-FU-based chemotherapy (98)	Partial patients received adjuvant chemotherapy	Non-restriction but the same inclusion criteria in the two arms	Bilateral LLND	LAR, APR, Hartmann, ISR, or TPE	C, D, E, F,
			TME alone	870	N/A	N/A				45-50.4 Gy/25 Gy + oxaliplatin-based/5-FU-based chemotherapy (870)			NO		

For matching criteria *A* = year, *B* = sex, *C* = tumor location, *D* = neo-adjuvant, *E* = adjuvant, *F* = lateral lymph-node status, *G* = lymph and vessel invasive *H* = tumor differentiation *I* = lateral lymph node status, *Ω* Astler-Coller staging system, *@* values are presented as the median (range), *FU* fluorouracil, *Gy LAR* low anterior resection, *APR*A abdominoperineal resection, *Hartmann* Hartmann’s procedure

^a^Values are presented as the median (IQR)

^b^Values are presented as the mean ± standard deviation

**Table 3:** Results of meta-analysis comparing TME + LLND versus TME alone

	Number of studies	TME + LLND patients	TME patients	Total patients	HR/RR/WMD (95% CI)	*P* value	Study heterogeneity			
							*χ*^2^	df	*I*^2^	*P* value
Survival										
5-year survival	4	1088	1101	2189	0.93^a^(0.71–1.22)	0.62	6	3	50%	0.11
5-year disease-free survival	5	868	684	1552	0.99^a^(0.74–1.34)	0.96	9.93	5	50%	0.08
Recurrence										
Total recurrence	4	653	454	1107	0.98(0.81–1.18)	0.83	2.37	4	0%	0.67
Local recurrence	7	1290	1930	3220	0.71(0.56–0.89)	0.003	9.22	7	24%	0.24
Lateral recurrence	3	773	1596	2369	0.49(0.18–1.28)	0.14	5.87	2	66%	0.05
Distant recurrence	5	615	1204	1819	0.95(0.68–1.34)	0.78	8.84	5	43%	0.12
Peri-operative outcomes										
Length of operation (min)	4	716	479	1195	97.03^b^(75.35–118.72)	*P* < 0.001	82.14	3	96%	*P* < 0.001
Blood loss (mL)	4	716	479	1195	303.20^b^(156.82–449.58)	*P* < 0.001	201.99	3	99%	*P* < 0.001
Peri-operative mortality	2	578	414	992	1.52(0.18–12.65)	0.7	0.47	1	0%	0.49
Postoperative complications	3	578	414	992	1.35(1.05–1.74)	0.02	0.53	2	0%	0.77
Functional outcomes										
Urinary dysfunction	2	374	372	746	1.44(0·63–3.28)	0·38	4.93	1	80%	0.03
Sexual dysfunction	2	108	92	200	1.41(0.87–2.31)	0·17	2.23	1	55%	0.13

*HR* hazard ratio, *RR* risk ratio, *WMD* weighted mean difference, *df* degrees of freedom

^a^HR

^b^WMD

### Primary endpoints: 5-year OS and DFS

**Fig. 2:**
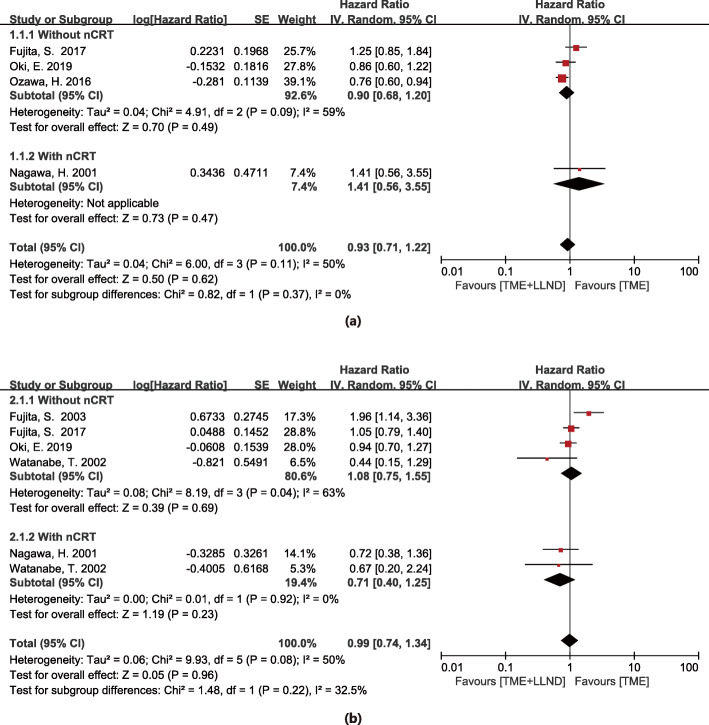
Total mesorectal excision and lateral lymph node dissection versus total mesorectal excision alone in 5-year overall survival (**a**), and 5-year disease-free survival (**b**); nCRT neoadjuvant chemoradiotherapy

Four studies, with a total of 2189 patients, were pooled into the analysis of 5-year OS [9–12]. The results demonstrated no significant difference in 5-year OS between the LLND group and TME alone group (HR 0.93, 95% CI 0.71–1.22, *P* = 0.62) with moderate heterogeneity (*I*^2^ = 50%, *P* = 0.11). Subgroup analysis showed no significant difference in 5-year OS between the two groups no matter nRCT used or not (HR = 1.41, 95% CI 0.56–3.55, *P* = 0.47 vs HR = 0.90, 95% CI 0.68–1.20, *P* = 0.42). The details are shown in Fig. 2a.

Five studies of 1552 patients, were pooled into the analysis of 5-year DFS [2, 9, 11, 12, 22]. The results have no significant difference in 5-year DFS between the two groups (HR 0.99, 95% CI 0.74–1.34, *P* = 0.96) with moderate heterogeneity (*I*^2^ = 50%, *P* = 0.08) between-studies. Subgroup analysis showed no significant difference in 5-year DFS between these two groups regardless of the application of nCRT (HR=0.71, 95% CI 0.40–1.25, *P* = 0.23 vs HR = 1.08, 95% CI 0.75–1.55, *P* = 0.69). The details are shown in Fig. 2b.

### Secondary endpoints: total, local, lateral, and distant recurrence, operation time, intraoperative blood loss, postoperative complications, perioperative mortality, sexual, and urinary dysfunction

**Fig. 3:**
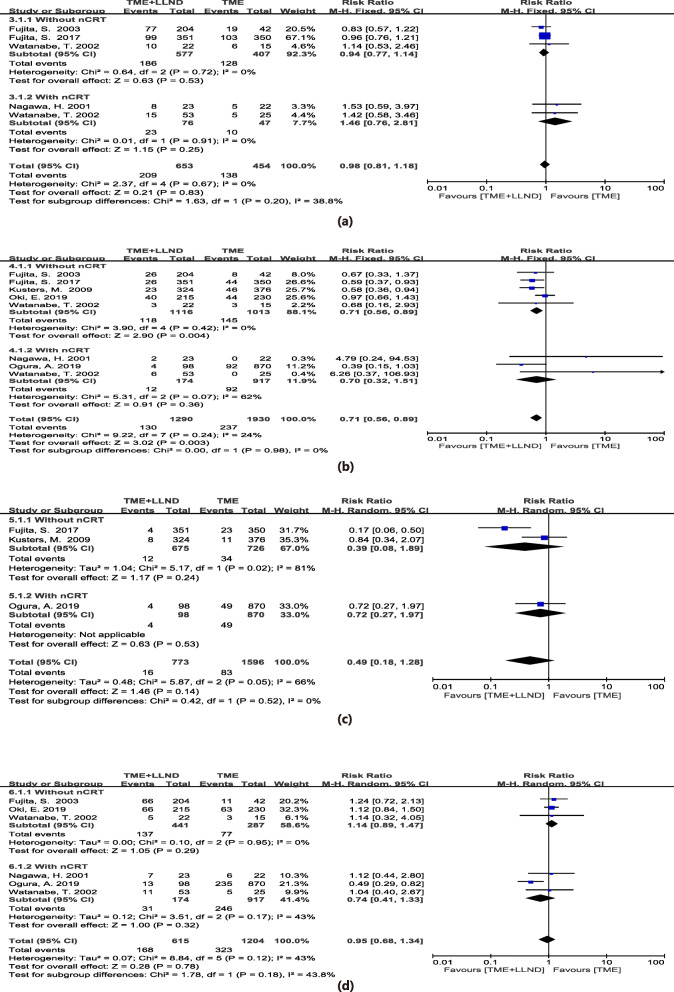
Total mesorectal excision and lateral lymph node dissection versus total mesorectal excision alone in 5-year total recurrence (**a**), local recurrence (**b**), lateral recurrence (**c**), and distant recurrence (**d**); nCRT neoadjuvant chemoradiotherapy

Four studies with a total of 1107 patients were eligible for the analysis of 5-year total recurrence [2, 9, 11, 22]. No significant difference in total recurrence was found between the two LLND and TME groups (RR 0.98, 95% CI 0.81–1.18, *P* = 0.83) with no heterogeneity (*I*^2^ = 0%, *P* = 0.67) between-studies. Subgroup analysis showed no significant difference in 5-year DFS between the two groups regardless of the application of nCRT (RR = 1.46, 95% CI 0.76–2.81, *P* = 0.25 vs RR = 0.94, 95% CI 0.77–1.14, *P* = 0.53). The details are shown in Fig. 3a.

Seven studies with a total of 3220 patients were pooled into the analysis of 5-year local recurrence [2, 6, 9, 11, 12, 16, 22]. The results indicated the LLND group had significantly lower 5-year local recurrence than the TME alone group (RR 0.71, 95% CI 0.56–0.89, *P* = 0.003) with low between-study heterogeneity (*I*^2^ = 24%, *P* = 0.24). Subgroup analysis found the LLND group had a significantly lower incidence of local recurrence than the TME alone group when preoperative nCRT was not performed (RR 0.71, 95% CI 0.56–0.89, *P* = 0.004). However, the difference was not significant once nCRT was introduced (RR 0.70, 95% CI 0.32–1.51, *P* = 0.36). The details are shown in Fig. 3b.

Data on 5-year lateral recurrences were extracted from 3 studies with 2369 patients [6, 9, 16]. The results demonstrated no significant difference in lateral recurrence between the two groups (RR 0.49, 95% CI 0.18–1.28, *P* = 0.14) with moderate heterogeneity (*I*^2^ = 66%, *P* = 0.05). Subgroup analysis indicated no significant difference in lateral recurrence between the two groups regardless of the introduction of nCRT (RR = 0.72, 95% CI 0.27–1.97, *P* = 0.53 vs RR = 039, 95% CI 0.08–1.89, *P* = 0.24). The details are shown in Fig. 3c.

Over 5-year distant recurrence was reported in 5 studies that investigated 1819 patients [2, 11, 12, 16, 22]. The results demonstrated no significant difference in distant recurrence between the two groups (RR 0.95, 95% CI 0.68–1.34, *P* = 0.78) with moderate heterogeneity (*I*^2^ = 43%, *P* = 0.12). Subgroup analysis revealed no significant difference in distant recurrence between the two groups regardless of the application of nCRT (RR = 0.74, 95% CI 0.41–1.33, *P* = 0.32 vs RR = 1.14, 95% CI 0.89–1.47, *P* = 0.29). The details are shown in Fig. 3d.

**Fig. 4:**
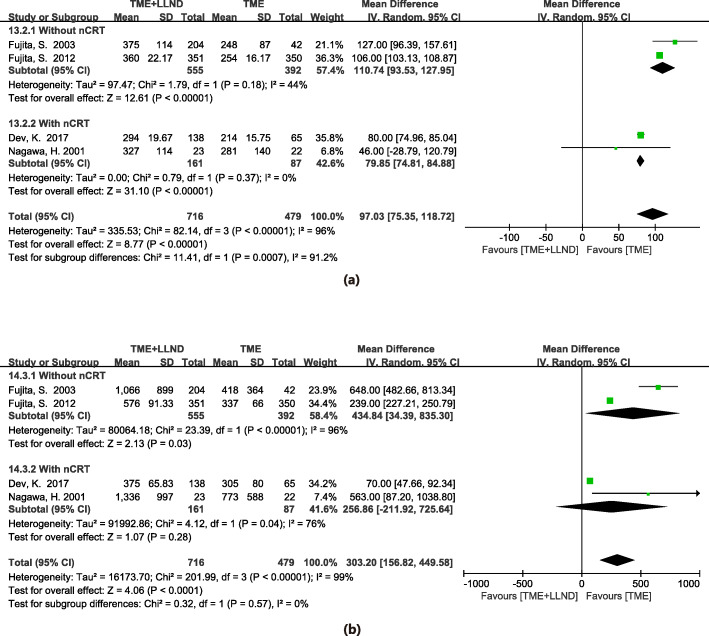
Total mesorectal excision and lateral lymph node dissection versus total mesorectal excision alone in operation time (**a**), and intraoperative blood loss (**b**); nCRT neoadjuvant chemoradiotherapy

Four studies were included in the meta-analysis that assessed the length of operation in 1195 patients [2, 11, 15, 23]. Results demonstrated a significant difference that favored the TME alone group (WMD 90.73 min, 95% CI 75.35–118.72, *P* < 0.001) with heterogeneity (*I*^2^ = 96%, *P* < 0.001). The details are shown in Fig. 4a.

Four studies were included in the meta-analysis to assess intraoperative blood loss in 1195 patients [2, 11, 15, 23]. Results indicated the TME alone group showed significantly lower intraoperative blood loss than the LLND group (WMD 303.20 mL, 95% CI 156.82–449.58, *P* < 0.001) with high heterogeneity (*I*^2^ = 99%, *P* < 0.001). The details are shown in Fig. 4b.

**Fig. 5:**
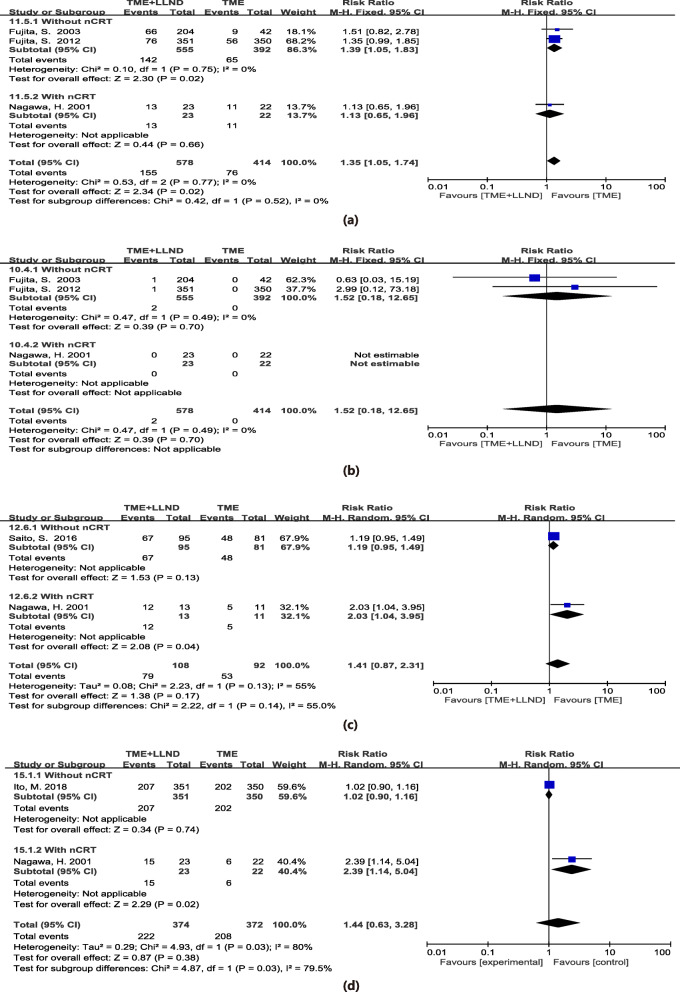
Total mesorectal excision and lateral lymph node dissection versus total mesorectal excision alone in postoperative complications (**a**), perioperative mortality (**b**), postoperative sexual dysfunction (**c**), and postoperative urinary dysfunction (**d**); nCRT neoadjuvant chemoradiotherapy

Three studies assessed 992 patients and reported postoperative complications [2, 11, 23]. The LLND group was associated with a higher rate of postoperative complications than the TME alone group (RR = 1.35, 95% CI 1.05–1.74, *P* = 0.02) with no heterogeneity (*I*^2^ = 0%, *P* = 0.77). The details are shown in Fig. 5a.

Perioperative mortality was reported in three studies that investigated 992 patients [2, 11, 23]. The data extracted from one of the studies were not suitable for meta-analysis because no events were mentioned in either group [11]. Ultimately, two studies, including 947 patients, were pooled into analysis [2, 23]. The results indicated no significant difference in perioperative mortality between the two groups (RR = 1.52, 95% CI 0.18-12.65, *P* = 0.70) with no heterogeneity (*I*^2^ = 0%, *P* = 0.49). The details are shown in Fig. 5b.

Two RCTs studies assessed 200 patients reported sexual dysfunction [11, 24]. Results indicated no significant difference in sexual dysfunction between two groups (pooled RR 1.41, 95% CI 0.87–2.31, *P* = 0.17) with moderate heterogeneity (*I*^2^ = 55%, *P* = 0.13). The details are shown in Fig. 5c.

Two RCTs studies of 746 patients assessed and reported urinary dysfunction [11, 25]. Our results demonstrated no significant difference in urinary dysfunction between the two groups (pooled RR 1.44, 95% CI 0.63–3.28, *P* = 0.38) with high heterogeneity (*I*^2^ = 80%, *P* = 0.03). The details are shown in Fig. 5d.

## Discussion

This meta-analysis is to assess the efficiency and safety of LLND in stage II/III of lower rectal cancer. The study demonstrates LLND reduced the local recurrence significantly without any considerable impact on distant cancer recurrence. Nevertheless, it has no advantage in increasing the rate of survival irrespective of nCRT use.

LLND showed significantly reduced local recurrence in patients who did not receive nCRT. This is neither posed additional risk of postoperative mortality nor increase the risk of sexual and urinary dysfunction. Our results do vary from previous meta-analyses performed by Georgiou et al. and Chen et al. a decade ago [13, 14]. Their studies suggested LLND neither reduced tumor recurrence nor prolonged survival time but significantly affected urinary and sexual function. However, the limited quality of the studies is included in analyses, inherent flaws in their results. For example, the clinical characteristics were significantly different between the two groups, and the LLND group had more advanced tumors, i.e., larger (higher T stage) [26], node-positive [26, 27], and more aggressive pathology [28] compared to the TME alone group. Furthermore, upper rectal cancers and early-stage rectal tumors (T1) were also included in their studies [28–32]. However, the Japanese guidelines have recommended the application of LLND limited to stage II/III of lower rectal cancer regardless of lymphatic node metastasis [4]. Besides this, in the study performed by Chen and colleagues, the time-to-event data were analyzed as dichotomous outcomes instead of the generally recommended method of log HRs and its standard error [14]. Therefore, there were certain limitations in applying their results to guide the application of LLND in clinical practice. Our study included more high-quality trials than the previous two meta-analyses. Therefore, our study provides more powerful and valid results than the previous two meta-analyses.

Our results demonstrated LLND significantly reduced local recurrence of the patients who didn't receive nCRT, but the difference was not significant when nCRT was performed. These results indicated the advantage of LLND in controlling local recurrence might be replaced with nCRT. Caution should be taken when interpreting these results because no subgroup analysis was performed based on the pretreatment size of LLNs. Previous studies indicated patients with positive LLNs have a higher rate of local recurrence. TME followed by nCRT was not sufficient for radical eradication of the metastatic LLNs to avoid local recurrence [33, 34]. Akiyoshi et al. reported 30-40% of patients with positive LLN developed local recurrence even after nCRT, and it reduced to almost zero when additional LLND was performed [35]. Ogura et al. also noted that 25.6% of patients with positive LLN developed local recurrence even after receiving nCRT and radical resection, and reduced to 5.7% when extra LLND was performed [16]. Therefore, for patients with positive LLNs who undergo nCRT followed by TME alone may not be sufficient, and selective LLND should probably be considered [7, 33]. The value of selective LLND in patients who received nCRT remains controversial. A current phase III Chinese randomized controlled trial (NCT02614157) to demonstrate the safety and efficacy of selective LLND after nCRT in the treatment of advanced lower rectal cancer-bearing enlarged LLNs is being performed and may provide more reliable evidence [36].

The pattern of local recurrence can be divided into three categories: central pelvis recurrence, anastomosis recurrence, and lateral recurrence. The current study found LLND reduced the incidence of lateral recurrence, but without significant difference. The reason may be the different local recurrence patterns in the studies. Several studies have shown that the most common site of local recurrence varies among patients of different geographical regions. A Dutch trial indicated the most common site of recurrence was the central pelvis, and only 24% of the local recurrence originated from the lateral pelvis in the TME alone group [6]. Further, a study from Sweden also demonstrated lateral recurrence was not a major cause of local recurrence, and only 6% (2/33) of patients with local recurrence exhibited lateral pelvic recurrence [37]. However, a study by Nagasaki et al. from Japan suggested the most common site of local recurrence was the lateral pelvis, and approximately 50% of the patients with local recurrence developed lateral recurrence [38]. In addition, a study by Kim et al. from Korea also demonstrated approximately 65% (42/65) of patients with local recurrence developed lateral pelvic recurrence even after receiving nCRT and radical dissection [39]. Analogously, Fujita et al. reported a much higher rate of lateral pelvic recurrence (57%) in the TME alone group than Kusters and colleagues (24%) in the current meta-analysis, which may be the reason for high heterogeneity in the study [6, 9]. Therefore, patients in East Asia tend to have a higher incidence of lateral pelvic recurrence, and LLND may play a more important role in East Asian patients than patients in Europe.

We also found that LLND couldn't improve the overall 5-year survival or DFS of patients with rectal cancer regardless of the application of nCRT. The results indicate LNNs metastases might be a sign of systemic disease with a poor prognosis rather than a regional disease and couldn't be eliminated by surgery only [5]. Previous studies have demonstrated that the 5-year OS of patients with lateral lymph node enlargement is still poor (20–45%) even though local control has been achieved by the application of LLND [1, 40, 41]. Oki et al. also indicated LLND brings no benefits in improving 5-year DFS or OS of patients who did not receive nCRT [12]. Besides, the Japanese randomized trial also demonstrated LLND could not prolong the survival time of patients with rectal cancer [9]. The latest tumor node metastasis (TNM) classification by AJCC 8th edition classification LLNs involvement as distant disease, and TME followed by nCRT has been recommended as the standard treatment regimens [42]. However, whether LLND provides additional benefits to patients who have received nCRT remains controversial.

This meta-analysis result did not find any significant differences in 5-year OS and DFS between these groups after receiving nCRT. It is worth noting that these results were obtained without limiting the pretreatment size of LLNs and studies with negative LLNs were also included in our study. While, it has been reported that the pretreatment size of LLNs was significantly associated with survival outcomes, and patients with positive LLNs have significantly worse survival rates [39, 43]. MERCURY study demonstrated patients with enlarged LLNs had significantly lower 5-year DFS than that of the patients with negative LLNs (42% vs 70.7%) [43]. Kim et al. also identified LLNs short-axis diameter ≥ 10 mm was significantly associated with lower 5-year OS and DFS, even after nCRT and TME [39]. A subgroup analysis based on the pretreatment size of LLNs was planned during the design phase of the present meta-analysis, but no sufficiently detailed information was provided in the included trials to perform this subgroup analysis. Therefore, whether LLND provides additional survival benefits to patients with pretreatment-positive LLNs who received nCRT remained a mystery. A phase III Chinese randomized controlled trial (NCT02614157) may provide strong evidence on it [36].

Our data demonstrated TME followed by LLND required longer operation time and resulted in greater blood loss than TME alone. It is not difficult to understand that LLND combined with TME required more operation time because LLND is a meticulous procedure. Two trials performed around 2000 showed the mean difference in intraoperative blood loss was greater than 500 mL between the two groups [2, 11]. However, the recent two RCTs by Fujita et al. and Dev et al. showed the mean differences were just 239 mL and 70 mL, respectively [15, 23]. A reasonable explanation may be due to the improvements in surgical techniques, and blood loss may have been minimized compared with the earlier studies.

Our study also found that LLND was associated with more frequent and severe postoperative complications, however, did not increase the risk of postoperative mortality. These results should be interpreted with caution when applied to clinical practice, as all three trials included in the current meta-analysis reported extremely low incidences of postoperative mortality in both groups [2, 11, 23]. Notably, the aggregated data also demonstrated LLND did not bring additional risks to sexual or urinary dysfunction. Therefore, the potential damage to urinary and sexual function cannot be a stumbling block to prevent the application of LLND for the treatment of rectal cancer.

The limitations of the current study should not be neglected as a minimal number of RCTs were included, and four of the RCTs studies reported different outcomes based on the same randomized trial. Further, the results of another study were extracted from conference proceedings [15]. However, all of the included studies were of high quality (achieving more than seven stars) according to the Newcastle-Ottawa Scale (for non-RCTs) [44] or the Cochrane Collaboration's risk of bias tool (for RCTs) [45]. The follow-up times were different across studies, but the time was sufficient for outcomes to occur, and subjects lost to follow-up were unlikely to introduce bias. Despite meeting the inclusion criteria, clinical heterogeneity may present due to the different pretreatment statuses of LLNs between the included studies, which may have introduced bias. The chemoradiotherapy regimens, preoperative waiting time after neoadjuvant therapy, and specific surgical procedures and quality were different across studies, which presents another possibility to introduce bias. Despite these limitations, the current study provides the most comprehensive and up-to-date information on the frequently discussed value of the routine use of LLND in the treatment of stage II/III lower rectal cancer.

## Conclusion

This study demonstrates the advantage of LLND in locally advanced lower rectal cancer without using nCRT, even though it couldn't make much difference in OS or DFS in 5 years. However, risk of LLND is distressing when lateral lymph node's metastasis already occurred, and concern should be made on the longer operation time, greater blood loss, and other complications. Besides this, the well-controlled large numbers of RCT are needed to demonstrate whether selective LLND provides additional benefits over nCRT with LLND.

## Supplementary Information

**Additional file 1.** Risk of bias graph of RCTs. Review authors’ judgments about each risk of bias item presented as percentages across all included studies.

**Additional file 2.** Risk of bias summary of RCTs. Review authors’ judgments about each risk of bias item for each included study.

## Data Availability

No additional data is available.

## Abbreviations

TME: Total mesorectal excision
LLNs: Lateral pelvic lymph nodes
LLND: Lateral lymph node dissection
nCRT: Neoadjuvant Chemoradiotherapy
HR: Hazard ratio
RR: Relative risk
WMD: Weighted Mean Difference
OS: Overall survival
DFS: Disease-free survival
RCT: Randomised controlled trial
CI: Confidence interval
