# Supplementary figures and images for: Lateral lymph node dissection reduces local recurrence of locally advanced lower rectal cancer in the absence of preoperative neoadjuvant chemoradiotherapy: a systematic review and meta-analysis
Source: World J Surg Oncol. 2020 Nov 23;18:304. doi: 10.1186/s12957-020-02078-1 (PMC7685653; doi:10.1186/s12957-020-02078-1)

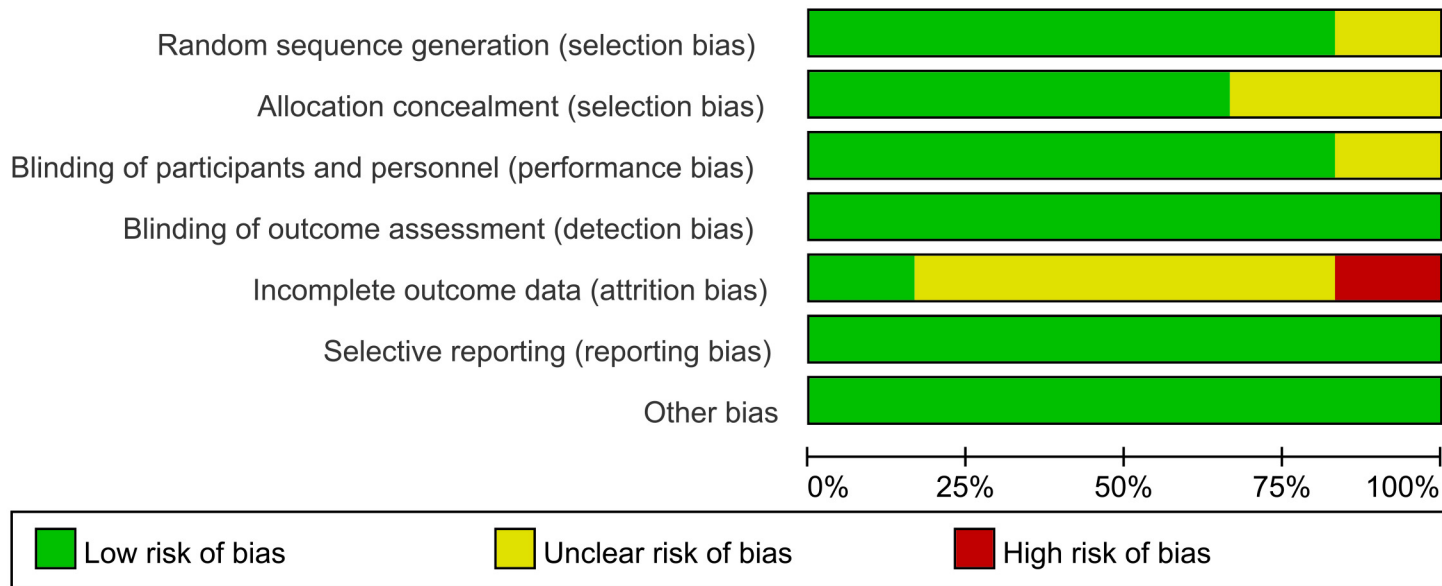

Supplement: Supplementary file 1 — Additional file 1. Risk of bias graph of RCTs. Review authors’ judgments about each risk of bias item presented as percentages across all included studies. [file 12957_2020_2078_MOESM1_ESM.pdf]
