# Supplementary material for: Lateral lymph node dissection reduces local recurrence of locally advanced lower rectal cancer in the absence of preoperative neoadjuvant chemoradiotherapy: a systematic review and meta-analysis
Source: World J Surg Oncol. 2020 Nov 23;18:304. doi: 10.1186/s12957-020-02078-1 (PMC7685653; doi:10.1186/s12957-020-02078-1)
Supplement: Supplementary file 2 — Additional file 2. Risk of bias summary of RCTs. Review authors’ judgments about each risk of bias item for each included study. [file 12957_2020_2078_MOESM2_ESM.pdf]

	Random sequence generation (selection bias)	Allocation concealment (selection bias)	Blinding of participants and personnel (performance bias)	Blinding of outcome assessment (detection bias)	Incomplete outcome data (attrition bias)	Selective reporting (reporting bias)	Other bias
Dev, K. 2017	+	?	?	+	−	+	+
Fujita, S. 2012	+	+	+	+	?	+	+
Fujita, S. 2017	+	+	+	+	?	+	+
Ito, M. 2018	+	+	+	+	?	+	+
Nagawa, H. 2001	?	?	+	+	+	+	+
Saito, S. 2016	+	+	+	+	?	+	+
